# Mechanism of RNA Oxidation and Its Inhibitor Involved in Ang II‐Induced Cardiomyocyte Hypertrophy

**DOI:** 10.1002/agm2.70057

**Published:** 2025-12-19

**Authors:** Tong Liu, Jin Bian

**Affiliations:** ^1^ Department of Cardiology Beijing Anzhen Hospital, Capital Medical University Beijing China; ^2^ Department of Liver Surgery, Peking Union Medical College Hospital Chinese Academy of Medical Sciences and Peking Union Medical College (CAMS & PUMC) Beijing China

**Keywords:** 8‐oxoG, cardiomyocyte hypertrophy, ERK–MAPK, RNA oxidation

## Abstract

**Objectives:**

To explore the mechanism of RNA oxidation and its inhibitor MTHI involved in cardiomyocyte hypertrophy.

**Methods:**

The hypertrophic H9c2 cardiomyocytes were stimulated with different concentrations and times of Ang II (Ang II) to construct a model of hypertensive heart failure in vitro. Transfection of H9c2 cells with the MTH1 overexpression plasmid was performed. The mRNA expression of ANP, BNP, and β‐MHC in each experimental group was detected by PCR. The expression of 8‐oxoG in H9c2 cells was determined by immunofluorescence and enzyme‐linked immunosorbent assay (ELISA). The activation of the ERK–MAPK pathway and the amount of MTH1 protein were detected by WB semi‐quantitative method.

**Results:**

Notably, RNA oxidation is a critical event in cellular senescence, and its accumulation is strongly linked to the aging process and the development of age‐related diseases. In our model of cardiomyocyte hypertrophy, the oxidative damage of RNA was aggravated, and the expression of MTH1 was increased. At the same time, the sequence of ERK–MAPK pathway proteins was activated. It can be seen that the oxidative damage of RNA is related to the process of cardiomyocyte hypertrophy. After transfection of the MTH1 overexpression plasmid into the cardiomyocyte hypertrophy model, we found that the amount of 8‐oxoG decreased, and the activation of ERK–MAPK signaling pathway proteins decreased, and H9c2 cell hypertrophy decreased. Therefore, we concluded that 8‐oxoG may aggravate the hypertrophy of the cardiomyocyte hypertrophy model by activating the ERK–MAPK pathway.

**Conclusion:**

The oxidative damage of RNA is involved in the process of cardiomyocyte hypertrophy. The mechanism may be that 8‐oxoG, a product of RNA oxidation, activates the downstream ERK–MAPK signaling pathway. These findings provide new perspectives for further exploration into the role of RNA oxidation in the pathogenesis of age‐related diseases, particularly heart failure.

## Graphical abstract

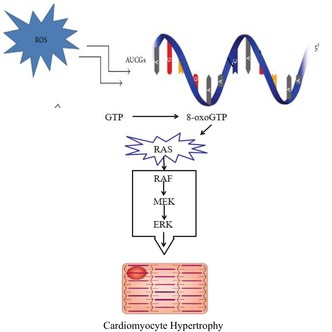

## 1 Introduction

Heart failure (HF) is a prototypical age‐related disease, whose incidence increases exponentially with advancing age. It represents a major global public health challenge, with end‐stage patients having a poor prognosis and a 5‐year mortality rate exceeding 50% [1]. According to the american heart association (AHA) 2023 statistical report, the prevalence of HF is approximately 7% among individuals aged 65–79 years and rises sharply to nearly 14% in the population aged 80 years and older [2], which strongly confirms its status as a highly prevalent disease among the elderly population. Among various etiologies, hypertension is a significant risk factor for HF, with approximately 40% of HF patients exhibiting ventricular diastolic dysfunction—a condition more prevalent in the elderly population [3]. Chronic hypertension increases cardiac afterload, leading to myocardial hypertrophy and fibrosis, which in turn causes ventricular remodeling. This pathological process reduces myocardial compliance and ultimately progresses to diastolic dysfunction heart failure [4].

Reactive oxygen species (ROS) induce oxidative damage to DNA and RNA, contributing to ventricular remodeling in heart failure (HF) [5, 6]. RNA is more susceptible to oxidation than DNA due to its single‐stranded structure, lack of protective proteins, and proximity to mitochondria—the primary source of ROS [7]. Consequently, oxidized RNA (e.g., 8‐hydroxyguanosine, 8‐oxoG) accumulates more extensively than damaged DNA [8]. 8‐oxoG is associated with age‐related diseases, including HF with preserved ejection fraction (HFpEF) [9, 10, 11]. Our clinical studies revealed elevated urinary 8‐oxoG in HFpEF patients, correlating with disease severity. Mechanistically, 8‐oxoG activates the Ras/Raf1/MEK/ERK pathway, promoting cardiomyocyte hypertrophy and apoptosis [12, 13, 14]. While mammalian cells express RNA oxidation inhibitors (MTH1/NUDT5) that scavenge oxidized nucleotides [15, 16], MTH1—though upregulated in tumors—remains poorly characterized in HF myocardium [15, 16].

Numerous studies have investigated the association between DNA oxidation and heart failure globally; however, the pathogenic role of RNA oxidation in heart failure remains poorly understood. As a significant biomarker of aging, 8‐oxoG has been confirmed to be markedly elevated in various age‐related diseases (such as neurodegenerative diseases and diabetes), yet its mechanistic role in heart failure, particularly HFpEF, remains to be systematically elucidated. Our research group is the first in China to systematically explore the relationship between RNA oxidation and heart failure. Specifically, we examined the role of RNA oxidation in the pathogenesis of HFpEF in elderly patients. Our analysis revealed significantly elevated levels of urinary 8‐oxoG in elderly HFpEF patients compared with the control group. Furthermore, immunohistochemical staining demonstrated higher 8‐oxoG expression in myocardial tissues from HFpEF patients than in those from non‐failure controls. Based on comprehensive medical record reviews and quantitative measurements of urinary 8‐oxoG, we preliminarily concluded that RNA oxidation levels are elevated in heart failure patients relative to healthy individuals, suggesting a potential involvement of RNA oxidation in the pathogenesis of heart failure. Nevertheless, the precise molecular mechanisms underlying RNA oxidation‐induced heart failure, as well as alterations in related RNA oxidation inhibitory enzymes, remain unclear. Research in this area represents a significant knowledge gap internationally. The H9c2 cell line is a subcloned cell of embryonic myocardium of bdlx rats. It has been widely used in the research of various heart diseases in the world. Given the role of RNA oxidation in promoting aging and related diseases, its function in the development and progression of HF warrants further investigation. In this study, we constructed an in vitro model of hypertension in H9c2. The expression of 8‐oxo‐G, cardiac hypertrophy index, and RNA oxidation inhibitor were measured. Meanwhile, the expression of ERK–MAPK pathway protein in the heart of each group was detected to explore the correlation between RNA oxidation and the pathogenesis of HF in vitro. RNA oxidation and its inhibition mechanism may be involved in the HF pathway.

## 2 Materials and Methods

### 2.1 Establishment and Grouping of Cell Models of Heart Failure

In the first part of the experiment, H9c2 cells stimulated by Ang II (Shanghai Cell Bank, Chinese Academy of Sciences) were used as the in vitro cell model to simulate human hypertensive heart failure, and the effect of Ang II on the expression of 8‐oxoG in H9c2 cells was detected. Firstly, H9c2 cells were treated with different concentrations of Ang II (10^−8^, 10^−7^, 10^−6^ M) for 24 h. The expression of 8‐oxoG in H9c2 cells was detected by cell immunofluorescence and ELISA, respectively. The change trend of 8‐oxoG expression in H9c2 cells of different groups was observed, and the concentration of Ang II required for maximal 8‐oxoG induction was determined. The experiment was divided into control group: H9c2 cells were incubated with DMEM containing 1% calf serum for 24 h; Ang II 10^−8^ M group: H9c2 cells were incubated with 10^−8^ M Ang II for 24 h; Ang II 10^−7^ M group: H9c2 cells were incubated with 10^−7^ M Ang II for 24 h; Ang II 10^−6^ M group: H9c2 cells were incubated with 10^−6^ M Ang II for 24 h. Secondly, we treated H9c2 cells with the optimal concentration of Ang II for 0, 12, 24, and 36 h respectively, and then detected the expression of 8‐oxoG in H9c2 cells by immunofluorescence and ELISA. We observed the change trend of 8‐oxoG expression in H9c2 cells of different groups and found out the ang that induced H9c2 cells to produce the most 8‐oxoG. The experiment was divided into 0 h group: H9c2 cells were incubated with DMEM containing 1% calf serum for 24 h; 12 h group: H9c2 cells were incubated with Ang II for 12 h; 24 h group: H9c2 cells were incubated with Ang II for 24 h; 36‐h group: H9c2 cells were incubated with Ang II for 36 h. Through the above two experiments, the optimal experimental concentration and time of Ang II stimulating H9c2 cells were found. The second part of the experiment: the experiment was divided into four groups: normal control group (Group C): normal H9c2 cells; experimental group (Group S): normal H9c2 cells + Ang II treatment group; overexpression group (Group G): normal H9c2 cells + MTH1 overexpression + Ang II treatment group; empty loading group (Group K): normal H9c2 cells + MTH1 empty loading + Ang II treatment group. The expression of 8‐oxoG was detected by immunofluorescence and ELISA, and the surface area of cells was measured. The mRNA expression of ANP, BNP, and β‐MHC was detected by real‐time PCR. The expression of ERK–MAPK and MTH1 was detected by WB. Through the above experiments, the possible mechanism of RNA oxidation and its inhibitor involved in Ang II‐induced cardiomyocyte hypertrophy was explored.

### 2.2 Production Process of MTH1 Plasmid

Design primer information: CMV‐F: CGC AAA TGG GCG GTA GGC GTG. The construction of the MTH1 plasmid employed a restriction enzyme digestion‐ligation cloning strategy. The cloning vector used was the pmcherry‐N1 mammalian expression vector, which contains a CMV strong promoter, an mCherry red fluorescent reporter gene, and a neomycin resistance selection marker. Fresh plasmid was extracted from the MTH1 vector plasmid liquid, followed by double digestion with corresponding restriction enzymes such as XhoI and EcoRI. The MTH1 target fragment containing homologous restriction sites and the linearized vector were subjected to primer annealing and T4 ligation reaction, after which the plasmids were transformed into competent cells. Positive transformants were preliminarily screened via the mCherry fluorescent reporter gene, and plasmids were extracted for restriction enzyme digestion verification. Several positive transformants were selected for sequencing identification (using CMV‐F primers and gene‐specific primers). After alignment confirmed that the base sequence of the vector was entirely consistent with the expected reference sequence, the bacterial strains were preserved, ultimately confirming the successful construction of the MTH1‐pmcherry‐N1 recombinant expression vector. According to the experimental method in paper [17], the plasmid vector was extracted.

### 2.3 Flow Chart of 8‐oxoG in Cells Detected by Immunofluorescence

The glass wafer was sterilized to make cell climbing slice. The cells were treated with 0.5% Triton X‐100 for 20 min before immunofluorescence. First antibody 15A3 was dripped from Abcam (dilution ratio 1:300). Observe and film under fluorescence microscope. The fluorescence value of 8‐oxoG was calculated by image pro Plus 6.0 software.

### 2.4 The Process of 8‐oxoG Detection by ELISA

The level of 8‐oxoG in H9c2 cells of each experimental group was determined by enzyme‐linked immunosorbent assay (ELISA) kit (STA‐325, Cell Biolabs, USA). First, a standard curve was generated using reference samples. Subsequently, total RNA was extracted from the cell samples using a commercial RNA extraction kit.

### 2.5 The Flow of Cell Surface Area Measurement and Statistics in Each Group

H9c2 cardiomyocytes were taken from six well plates of each experimental group, with 3 wells in each group. The morphology of cardiomyocytes was observed with a Nikon inverted microscope, and the surface area of 10 cardiomyocytes was measured in each field. We used IPP software to measure and record the cell surface area.

### 2.6 Detection of ANP, BNP and β‐MHC mRNA by Real Time PCR

Firstly, RNA was extracted from myocardial tissue, and the extraction was carried out according to the detailed steps of the kit instructions, as shown in the paper [18]. Then, the reverse transcription reaction was performed. ANP mRNA, BNP mRNA, β‐MHC mRNA, and GAPDH mRNA were amplified.

### 2.7 Western Blot Analysis

Performing WB experiments according to standard procedures. The following antibodies were used: anti‐Raf1 rabbit antibody (41383; SAB;1:1000 dilution), anti‐p‐Raf1 rabbit antibody (11204; SAB; 1:1000 dilution), anti‐MEK1/2 rabbit antibody (8727; CST; 1:1000 dilution), anti‐p‐MEK1/2 rabbit antibody (2338; CST; 1:2000 dilution), anti‐ERK1/2 rabbit antibody (4695; CST; 1:1000 dilution), anti‐p‐ERK1/2 rabbit antibody (4370; CST; 1:2000 dilution), anti‐MTH1 rabbit antibody (ab187531; abcam; 1:1000 dilution), and anti‐GAPDH mouse antibody (TA‐08; ZSGB‐BIO; 1:2000 dilution). The density of the signal was analyzed with the Image J software program (NIH). The experiment was repeated three times.

### 2.8 Statistical Analysis

The parameters are expressed as mean ± standard deviation (SD). All data analyses were performed using SPSS 20. Differences were considered significant for all analyses in this study when the probability value was < 0.050. Normal Distribution and Homogeneity of Variance were checked for all the data. Comparisons between the two divided groups were made by Student's *t*‐test. Pearson's or Spearman's correlation test was used for correlation analysis.

## 3 Results

### 3.1 The Effect of Ang II on the Expression of 8‐oxoG in H9c2 Cells

#### 3.1.1 Comparison of the Changes of 8‐oxoG Production in H9c2 Cells Induced by Ang II at Different Concentrations

**FIGURE 1:**
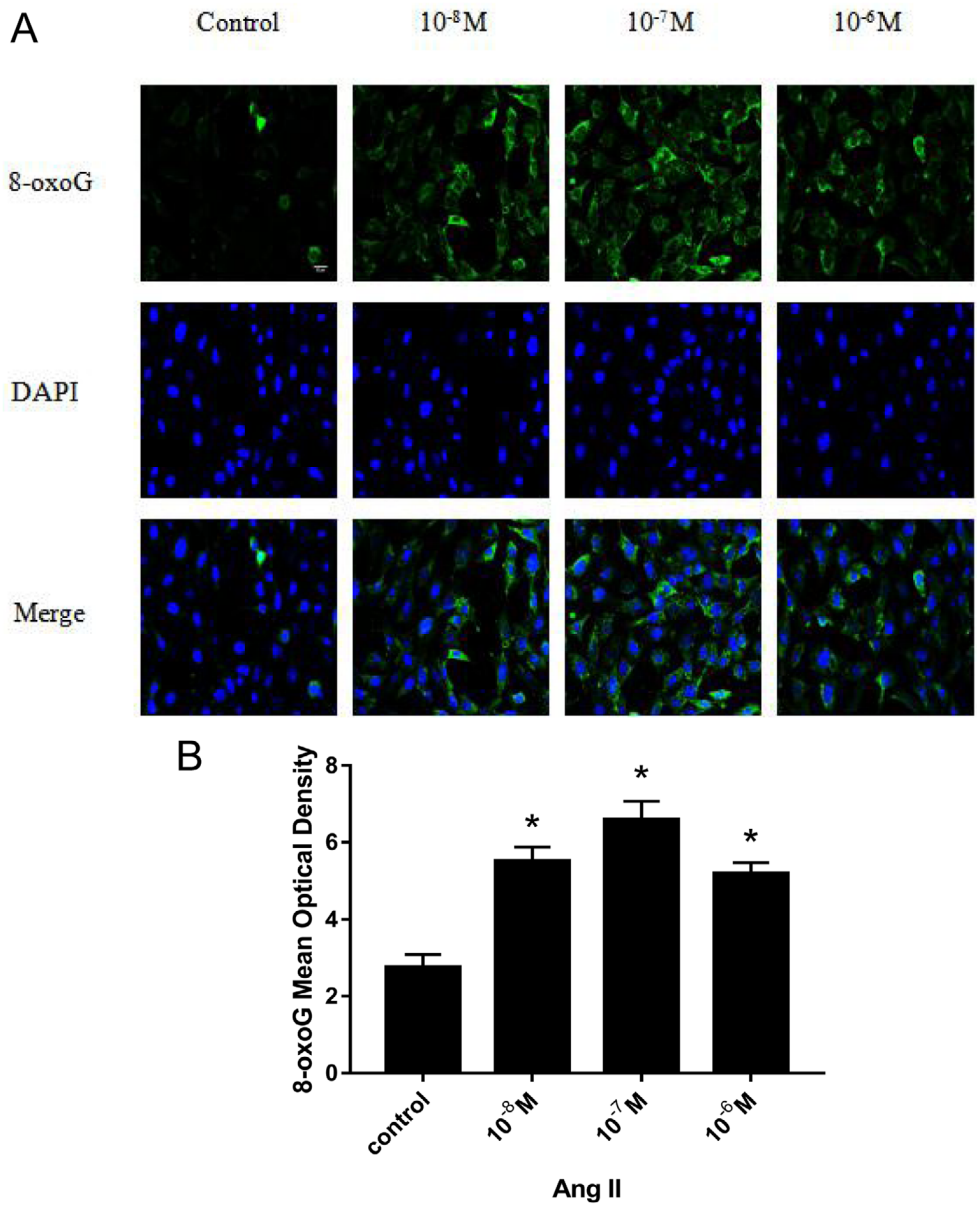
Comparison of the changes of 8‐oxoG production in H9c2 cells induced by different concentrations of Ang II for 24 h by immunofluorescence method. (A) is the immunofluorescence staining picture of 8‐oxoG produced by H9c2 cells in each group. (B) is a histogram of the mod value of 8‐oxoG produced by H9c2 cells in each group (the scale in the figure is 50 μm, $\bar{X}$ ± *s*, *n* = 6, *compared with the control group, the difference is statistically significant, *p < 0.01).

**FIGURE 2:**
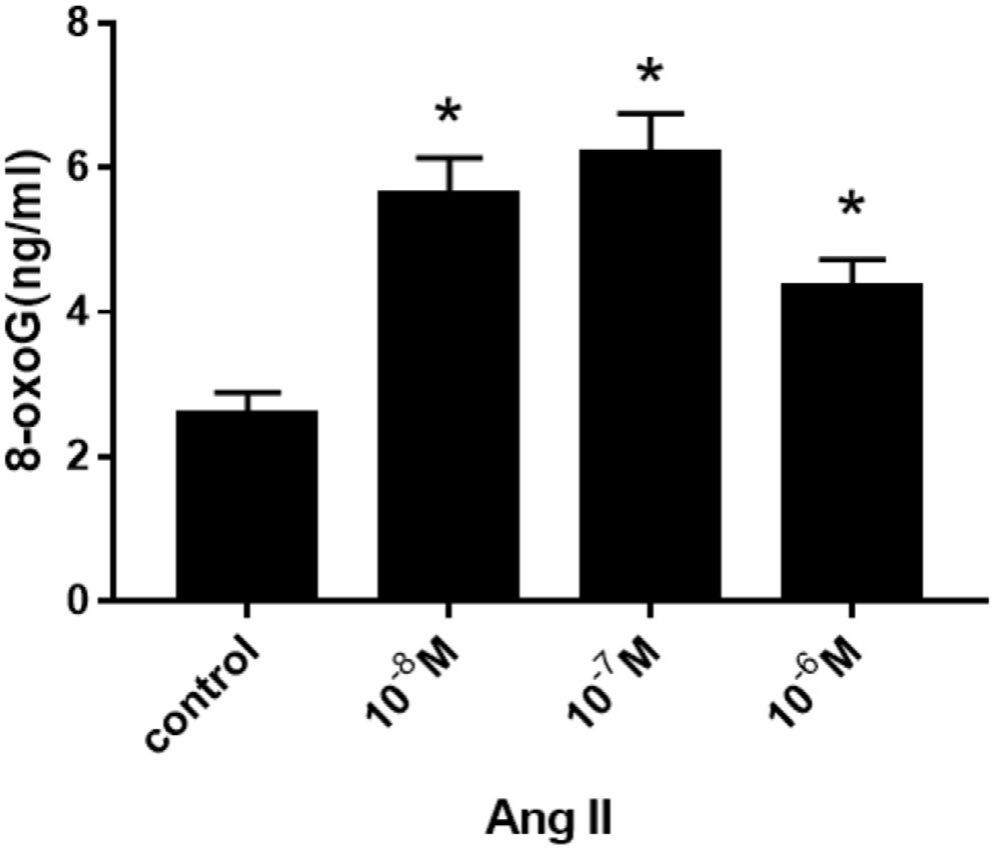
ELISA method was used to detect the change trend of 8‐oxoG expression in H9c2 cells induced by different concentrations of Ang II for 24 h ($\bar{X}$ ± *s*, *n* = 6, *compared with control group, the difference was statistically significant, *p < 0.01).

After H9c2 cells were treated with different concentrations of Ang II (10^−8^, 10^−7^, 10^−6^ M) for 24 h, the expression of 8‐oxoG in H9c2 cells was detected by immunofluorescence and ELISA, respectively. The change trend of 8‐oxoG expression in H9c2 cells in different groups was observed, and the optimal concentration of Ang II for inducing H9c2 cells to produce 8‐oxoG was found. Figure 1A is the immunofluorescence contrast picture of 8‐oxoG production in H9c2 cells among each group. The mean optical density (MOD) of 6 fluorescence microscopes in each group was used to represent the expression of 8‐oxoG. According to the statistical histogram in Figure 1B, the expression of 8‐oxoG in H9c2 cells treated with different concentrations of Ang II for 24 h increased in a concentration‐dependent manner. Compared with the control group, the increased amount in Ang II 10^−8^, 10^−7^, 10^−6^ M groups was statistically significant (*p < 0.001); among them, H9c2 cells produced the most 8‐oxoG in Ang II 10^−7^ M. Histogram 2 shows the change trend of 8‐oxoG (ng/mL) production in H9c2 cells detected by ELISA among Ang II concentration groups. We found that, similar to the changes measured by immunofluorescence, the expression of 8‐oxoG was increased in a concentration‐dependent manner by ELISA, and there was a significant difference between the Ang II 10^−8^, 10^−7^, 10^−6^ M groups and the control group (*p < 0.001); the amount of 8‐oxoG produced by H9c2 cells was the most in the Ang II 10^−7^ M group. According to the change trend of 8‐oxoG produced by H9c2 cells stimulated by different concentrations of Ang II, we found that the expression of 8‐oxoG was the highest at the concentration of Ang II 10^−7^ M, so we chose Ang II 10^−7^ M as the best concentration (Figure 2).

#### 3.1.2 Comparison of 8‐oxoG Production in H9c2 Cells Induced by Ang II at Different Times

**FIGURE 3:**
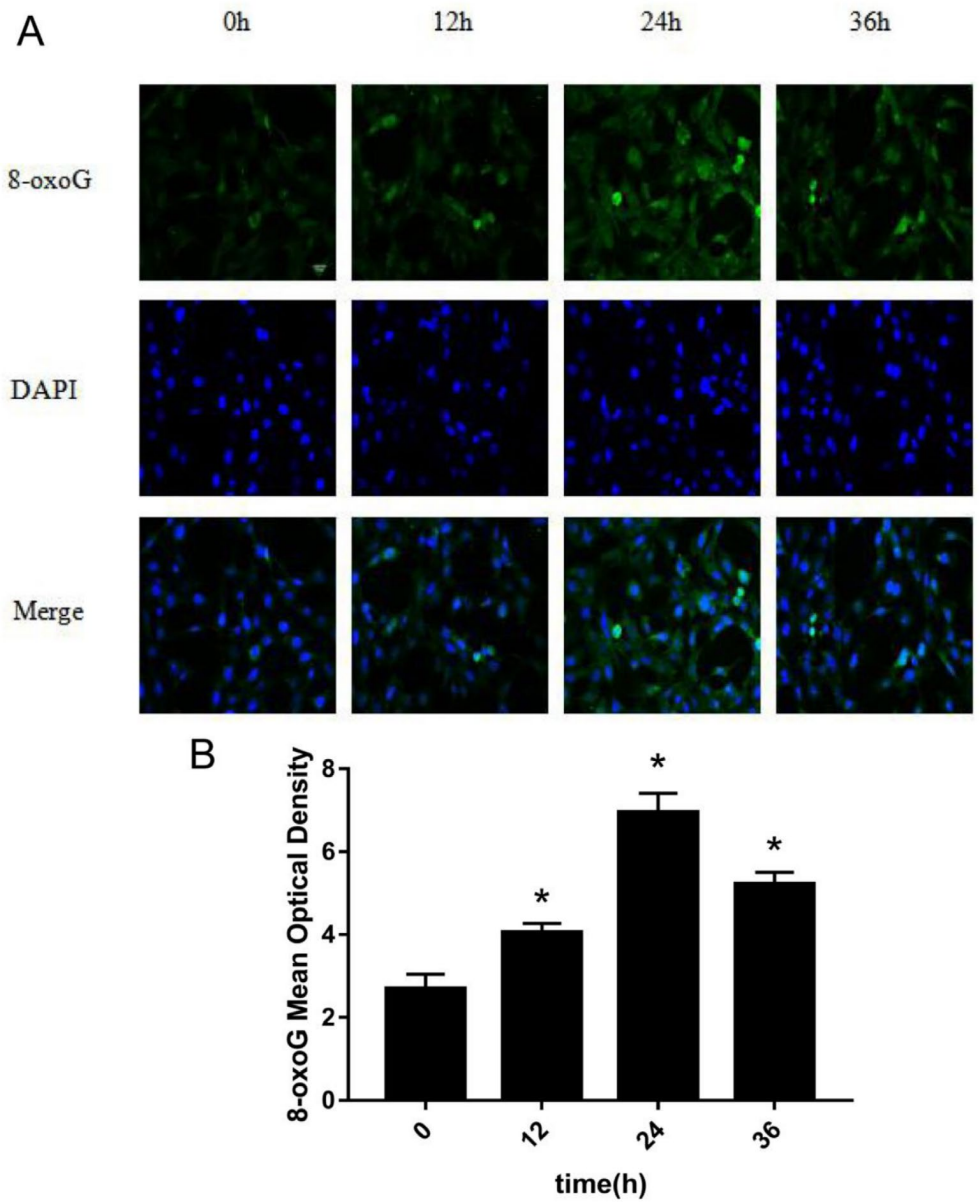
Comparison of 8‐oxoG production in H9c2 cells induced by Ang II 10^−7^ M at different time by immunofluorescence method. (A) is the immunofluorescence staining picture of 8‐oxoG produced by H9c2 cells in each group. (B) is a columnar statistical chart of the mod value of 8‐oxoG produced by H9c2 cells in each group (the scale in the figure is 50 μm, $\bar{X}$ ± *s*, *n* = 6, * compared with the 0‐h group, the difference is statistically significant, *p < 0.01).

**FIGURE 4:**
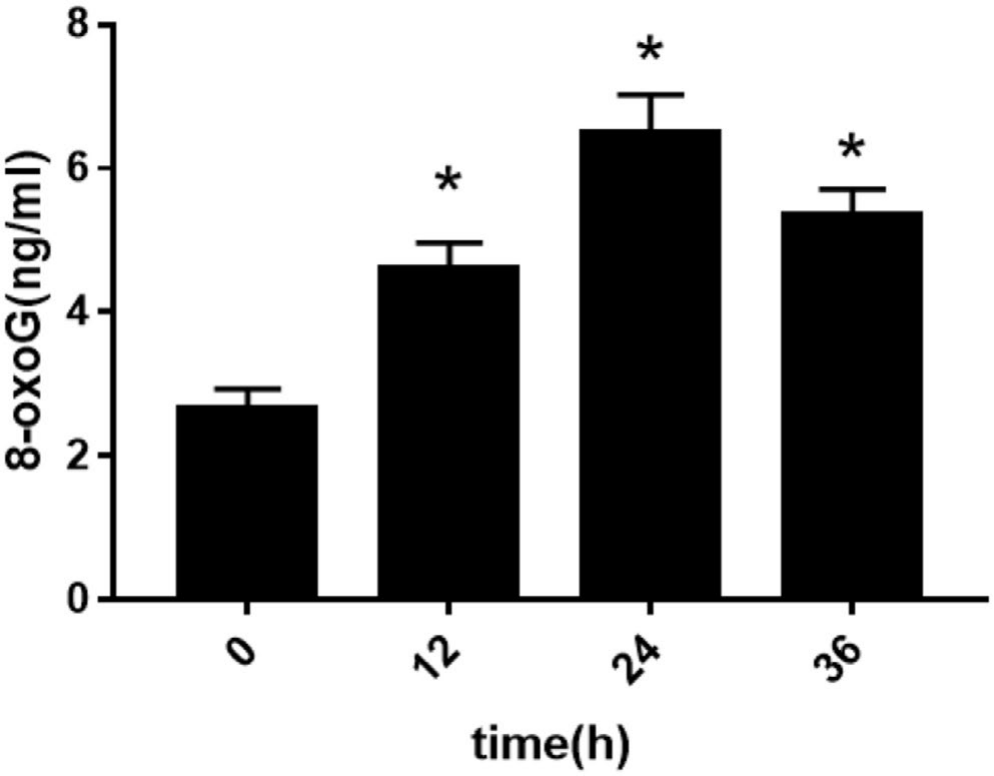
ELISA method was used to detect the change trend of 8‐oxoG expression in H9c2 cells induced by Ang II 10^−7^ M at different time ($\bar{X}$ ± *s*, *n* = 6, *compared with 0‐h group, the difference was statistically significant, *p < 0.01).

H9c2 cells were treated with 10^−7^ M Ang II for 0, 12, 24, and 36 h, respectively. The expression of 8‐oxoG in H9c2 cells was detected by immunofluorescence and ELISA. The change trend of 8‐oxoG expression in H9c2 cells of different groups was observed, and the optimal time for inducing H9c2 cells to produce 8‐oxoG was found. Figure 3A is the immunofluorescence contrast picture of 8‐oxoG production in H9c2 cells among each group. We used the software to count the mod of 6 fluorescence microscope high‐power fields in each group to represent the expression of 8‐oxoG. According to the statistical histogram in Figure 3B, the expression of 8‐oxoG in H9c2 cells treated with Ang II 10^−7^ M at different times increased in a time‐dependent manner. Compared with the 0 h group, the increase in the 12, 24, and 36 h groups of Ang II 10^−7^ M was statistically significant (*p < 0.001). Among them, H9c2 cells produced the most 8‐oxoG at 24 h of Ang II 10^−7^ M treatment. The column statistical Figure 4 describes the trend of the semi‐quantitative detection of 8‐oxoG (ng/mL) in H9c2 cells by ELISA between the 10^−7^ M time groups of Ang II. We found that the expression of 8‐oxoG was increased in a time‐dependent manner by ELISA, and the increase was statistically significant (*p* < 0.001) in the group of Ang II 10^−7^ M (12, 24, 36 h) and 0 h group (*p* < 0.001). We found that when Ang II 10^−7^ M stimulated cells for 24 h, the amount of 8‐oxoG produced by H9c2 cells was the most. The results of the changes of 8‐oxoG produced by Ang II 10^−7^ M stimulated H9c2 cells at different times were measured by the method of immunofluorescence and ELISA. We found that the expression of 8‐oxoG was the most at 24 h. Therefore, we take Ang II 10^−7^ M to stimulate H9c2 cells for 24 h as the best experimental time.

### 3.2 The Expression of 8‐oxoG in H9c2 Cells of Each Experimental Group Was Compared

**FIGURE 5:**
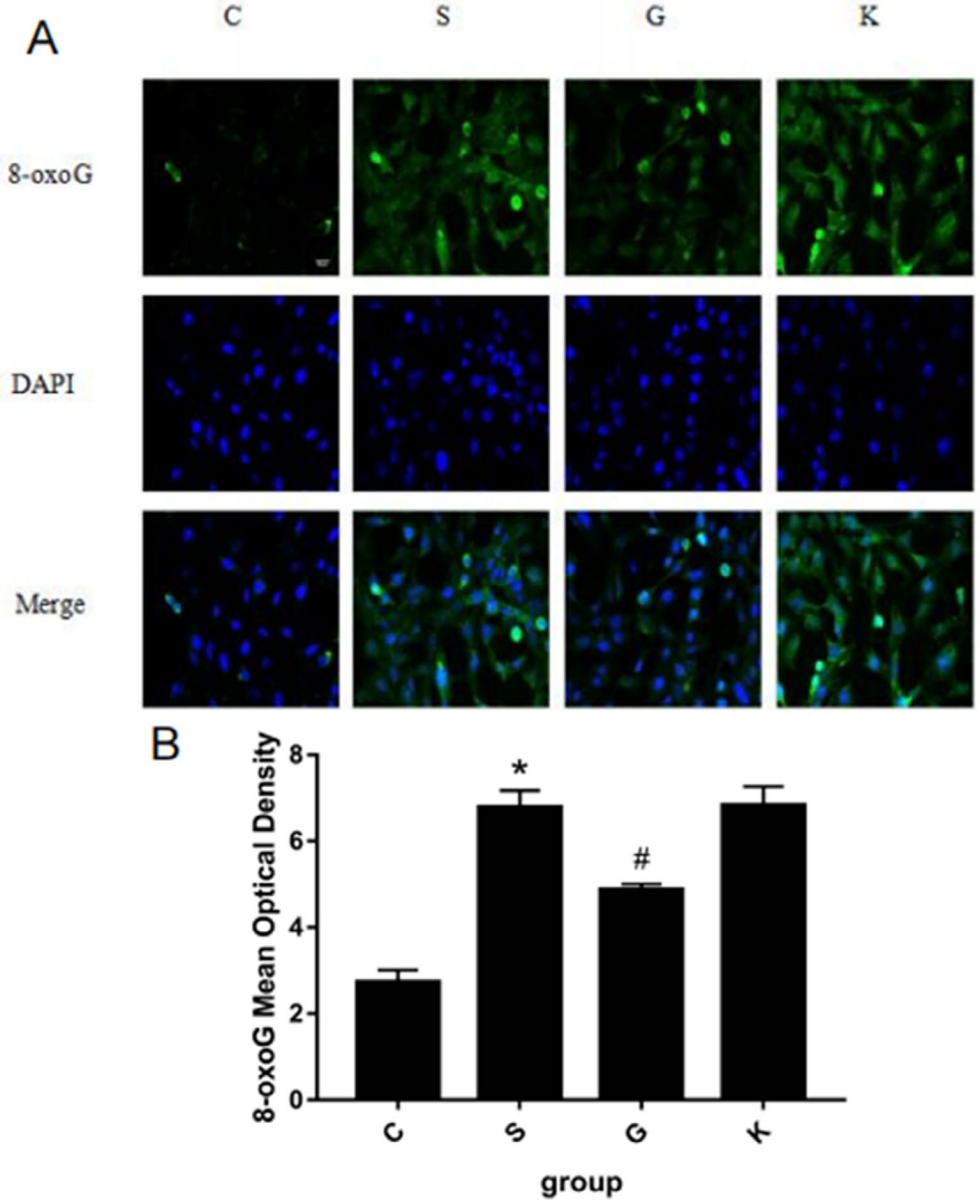
The expression of 8‐oxoG in H9c2 cells induced by Ang II 10^−7^ M was detected by immunofluorescence method for 24 h. (A) is the immunofluorescence staining picture of 8‐oxoG produced by H9c2 cells in each group. (B) is a histogram of the mod value of 8‐oxoG produced by H9c2 cells in each group (the scale in the figure is 50 μm, $\bar{X}$ ± *s*, *n* = 6, *S Group compared with C Group, the difference is statistically significant, *p < 0.01; ^#^G Group compared with S Group, the difference is statistically significant, ^#^ *p* < 0.01).

**FIGURE 6:**
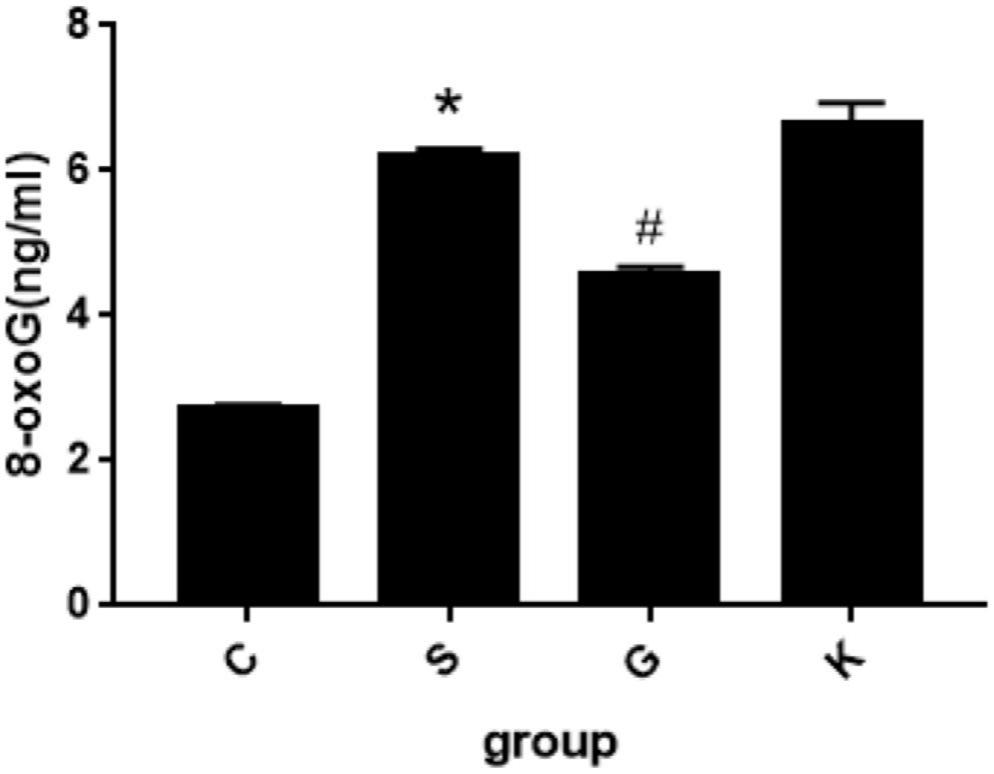
The expression of 8‐oxoG in H9c2 cells induced by Ang II 10^−7^ M was detected by ELISA for 24 h ($\bar{X}$ ± *s*, *n* = 6, *S Group compared with Group C, the difference was statistically significant, *p < 0.01; ^#^G Group compared with Group S, the difference was statistically significant, ^#^ *p* < 0.01).

Now we divided the experiment into four groups: normal control group (Group C): normal H9c2 cells, experimental group (Group S): normal H9c2 cells + ang II treatment group, overexpression group (Group G): normal H9c2 cells + MTH1 overexpression + Ang II treatment group, empty group (Group K): normal H9c2 cells + MTH1 empty + Ang II treatment group. Methods: H9c2 cells were treated with 10^−7^ M Ang II for 24 h. The expression of 8‐oxoG in H9c2 cells was detected by immunofluorescence and ELISA. Figure 5A is the immunofluorescence contrast picture of 8‐oxoG production in H9c2 cells among each group. We used the software to calculate the mod value of each group of 6 fluorescence microscope high‐power fields to represent the statistical expression of 8‐oxoG. According to the statistical histogram in Figure 6B, H9c2 cells in Group S were treated with Ang II 10^−7^ M. After 24 h of stimulation, the expression of 8‐oxoG was significantly increased compared with Group C (*p < 0.001); when MTH1 was overexpressed, the expression of 8‐oxoG in Group G was significantly decreased compared with Group S, the difference was statistically significant (*p < 0.001); however, the expression of 8‐oxoG in Group K with empty plasmid was similar to that in Group S, and there was no significant difference between the two groups. The difference was statistically significant (*p* > 0.050). Histogram 6 shows the change trend of 8‐oxoG (ng/mL) production in H9c2 cells by ELISA. We found that the changes in Group S were similar to those of immunofluorescence. After 24 h of stimulation, the expression of 8‐oxoG was significantly increased compared with that of Group C (*p < 0.001); when MTH1 was overexpressed, the expression of 8‐oxoG in Group G was significantly decreased compared with that in Group S (*p < 0.001); while the expression of 8‐oxoG in Group K with empty plasmid was similar to that in Group S, and there was no significant difference between the two groups. The difference was statistically significant (*p* > 0.050). The results showed that the content of 8‐oxoG in H9c2 cells stimulated by Ang II 10^−7^ M for 24 h was higher than that in the normal group; the overexpression of MTH1 could inhibit the production of 8‐oxoG; the expression of MTH1 in H9c2 cells stimulated by Ang II 10^−7^ M was higher than that in the normal group. The empty vector plasmid showed no significant effect on the production of 8‐oxoG in H9c2 cells stimulated with Ang II 10^−7^ M for 24 h.

### 3.3 The Changes of H9c2 Cell Hypertrophy in Each Experimental Group Were Compared

#### 3.3.1 Comparison of Surface Area of H9c2 Cells in Each Experimental Group

**FIGURE 7:**
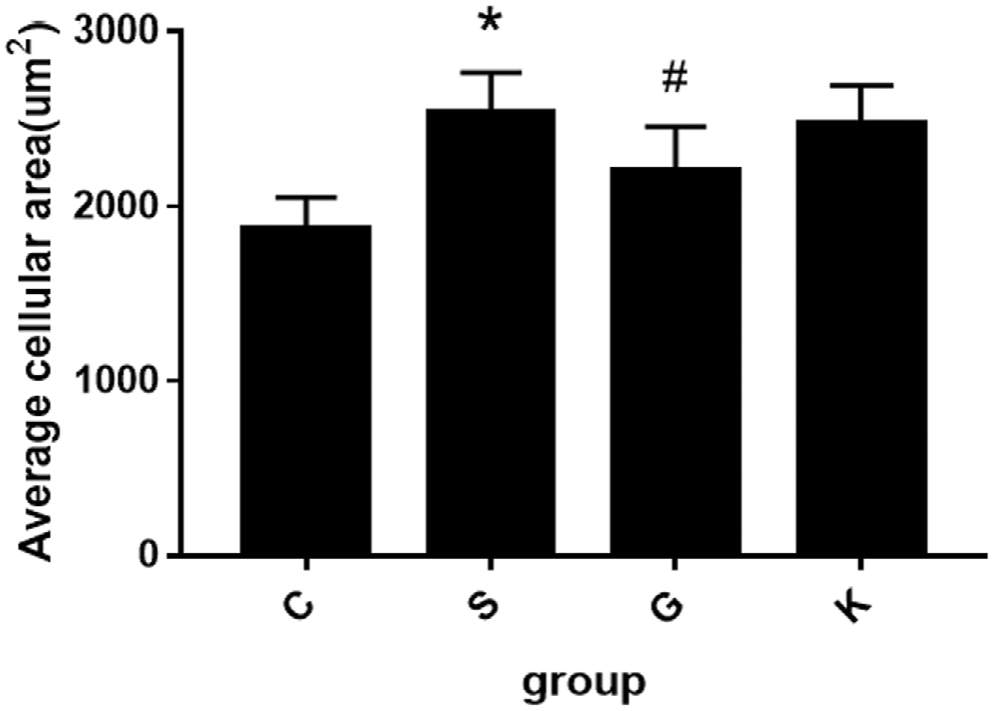
Comparison of cell surface area in each group ($\bar{X}$ ± *s*, *n* = 6, *S Group compared with Group C, the difference was statistically significant, *p < 0.01; ^#^G Group compared with Group S, the difference was statistically significant, ^#^ *p* < 0.05).

Under 400× light microscope, we took 6 cardiac myocyte fields in each group. The surface area of 10 H9c2 cells in each field was measured by IPP software. The statistics after taking the average value are shown in Figure 7 below. According to the histogram, H9c2 cells in Group S were treated with Ang II 10^−7^ M after 24 h stimulation, the cell surface area increased significantly compared with Group C (*p < 0.001); when the expression of MTH1 protein in cells was over expressed, the cell surface area of Group G was smaller than that of Group S, the difference was statistically significant (*p < 0.050); while the cell surface area of Group K and Group S with empty plasmid was the same, and there was no significant difference between the two groups. There was no statistical difference (*p* > 0.050).

#### 3.3.2 The Changes of ANP, BNP, and β‐MHC mRNA in H9c2 Cells of Each Experimental Group Were Compared

**FIGURE 8:**
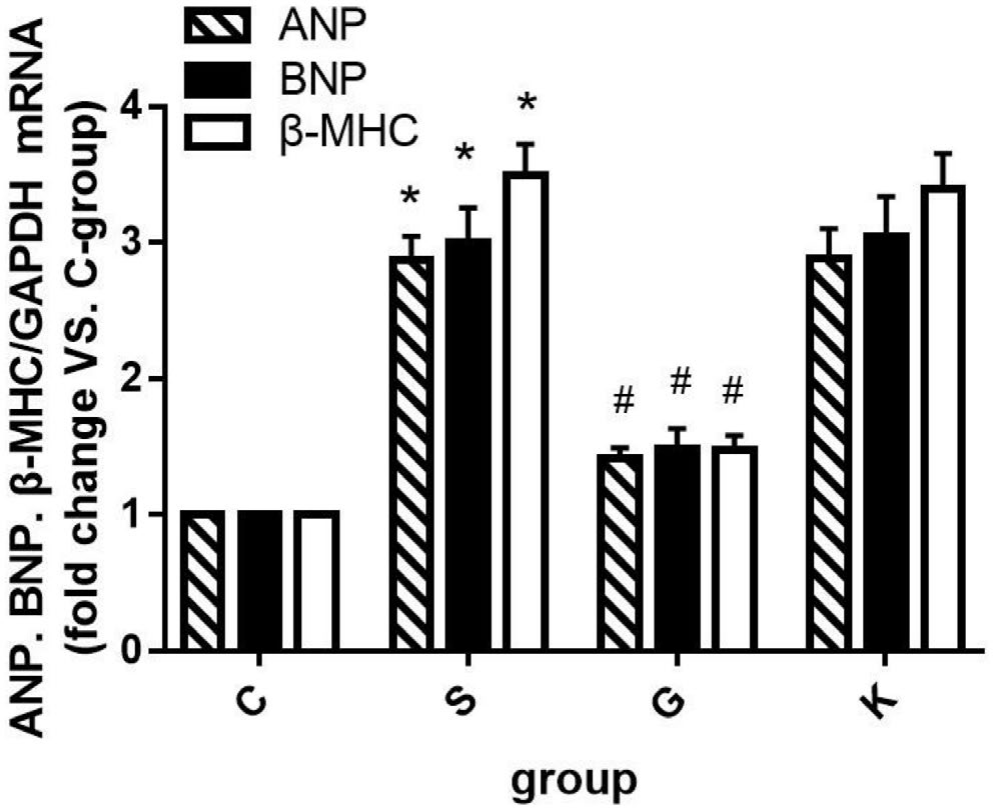
Comparison of the change trend of ANP, BNP, and β‐MHC mRNA in each group ($\bar{X}$ ± *s*, *n* = 3, *S Group compared with Group C, the difference was statistically significant, *p < 0.01; ^#^G Group compared with Group S, the difference was statistically significant, ^#^ *p* < 0.01).

We extracted the RNA of H9c2 cells with the kit, determined the mRNA expression levels of ANP, BNP, and β‐MHC by PCR, calculated the relative expression levels of ANP, BNP, and β‐MHC with Group C, and counted them in Figure 8. As can be seen from the figure, the mRNA expressions of ANP, BNP, and β‐MHC in H9c2 cells of Group S stimulated by Ang II 10^−7^ M for 24 h were significantly increased compared with those of Group C (*p < 0.001); when the protein of MTH1 was over expressed, the mRNA expressions of ANP, BNP, and β‐MHC in Group G were significantly higher than those in Group S. The expression of ANP, BNP, and β‐MHC mRNA in H9c2 cells of K Group and S Group was similar, but there was no significant difference between the two groups (*p* > 0.050).

### 3.4 The Changes of MTH1 Expression in H9c2 Cells of Each Experimental Group Were Compared

**FIGURE 9:**
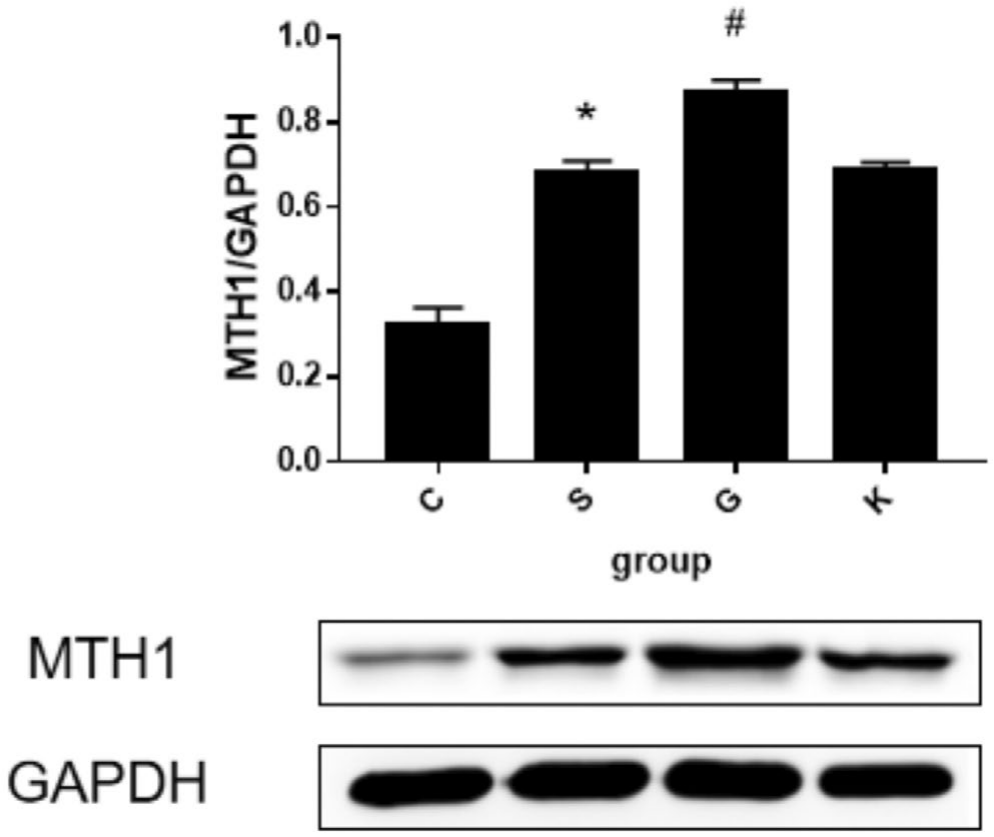
WB method was used to detect the change trend and band diagram of MTH1 protein expression in H9c2 cells of each experimental group. The protein expression was based on GAPDH. ($\bar{X}$ ± *s*, *n* = 3, *S Group compared with Group C, the difference was statistically significant, *p < 0.01; ^#^G Group compared with Group S, the difference was statistically significant, ^#^ *p* < 0.01).

We semi quantitatively detected the relative expression of MTH1 protein in H9c2 cells of each experimental group by WB method, as shown in Figure 9. From the histogram and WB banding map, we found that the H9c2 cells in Group S were treated with Ang II 10^−7^ M after 24 h of stimulation, the relative expression of MTH1 protein was significantly increased compared with Group C (*p < 0.001); when the expression of MTH1 protein was over expressed, the relative expression of MTH1 protein in Group G was significantly higher than that in Group S (*p < 0.001); in contrast, no statistically significant difference in MTH1 protein expression was detected between Group K (empty vector plasmid) and Group S (empty vector plasmid) in H9c2 cells (*p* > 0.050).

### 3.5 The Activation of ERK–MAPK Signal Transduction Pathway in H9c2 Cells of Each Experimental Group Was Compared

**FIGURE 10:**
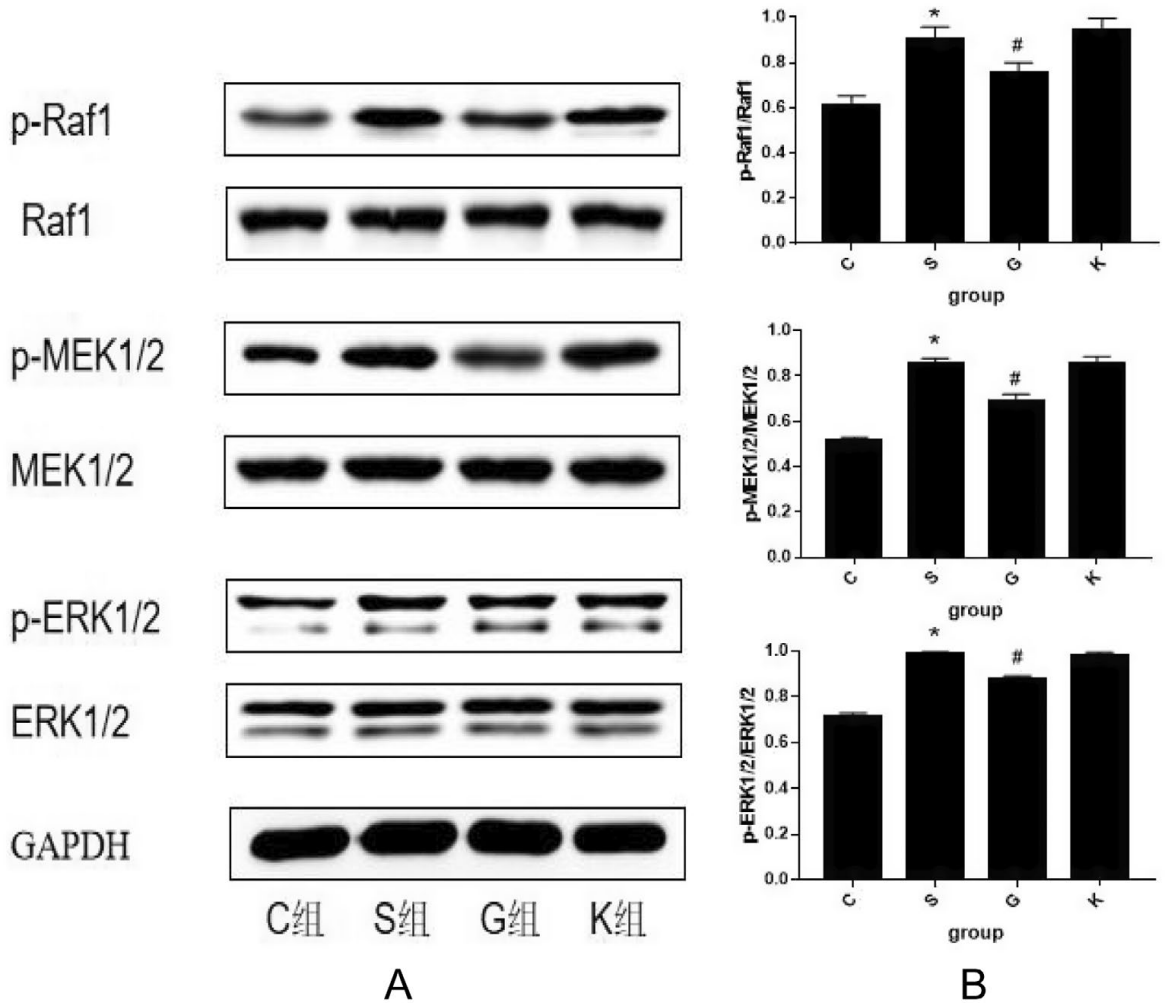
WB semi quantitative analysis of ERK–MAPK signaling pathway protein expression changes. (A) is the WB band diagram of ERK–MAPK pathway. (B) is a semi quantitative analysis of ERK–MAPK signaling pathway protein expression histogram. The protein expression was based on GAPDH ($\bar{X}$ ± *s*, *n* = 3, *S Group compared with Group C, the difference was statistically significant, *p < 0.01; ^#^G Group compared with Group S, the difference was statistically significant, ^#^ *p* < 0.05).

We used WB method to semi quantitatively analyze the ERK–MAPK pathway protein in H9c2 cells of each experimental group. The results of WB histogram and band are shown in Figure 10A,B. After data analysis and comparison, we believe that the H9c2 cells in Group S were treated with Ang II 10^−7^ M 24 h after stimulation, the values of p‐Raf1/Raf1, p‐MEK1/2/MEK1/2, p‐ERK1/2/ERK1/2 increased significantly compared with Group C (*p* < 0.001); when the protein of MTH1 was over expressed, the expression of p‐Raf1/Raf1, p‐MEK1/2/MEK1/2, p‐ERK1/2/ERK1/2 in Group G decreased compared with Group S (*p* < 0.001). However, the expression levels of 的p‐Raf1/Raf1, p‐MEK1/2/MEK1/2, p‐ERK1/2/ERK1/2 in H9c2 cells of Group K and Group S were similar, and there was no significant difference between the two groups (*p* > 0.050) (Figure 10A).

## 4 Discussion

Based on our earlier animal studies [19], this experiment explored the molecular mechanism of RNA oxidation and MTH1 involved in cardiomyocyte hypertrophy in vitro. Our study found that the expression of MTH1, an inhibitor of RNA oxidation, and the ERK–MAPK pathway was activated in the H9c2 cardiomyocyte hypertrophy model induced by Ang II. After overexpression of MTH1 in H9c2 cardiomyocytes, we found that the level of RNA oxidation was significantly decreased, and the activation of the ERK–MAPK pathway was decreased, accompanied by the decrease of cardiomyocyte hypertrophy.

Both primary rat cardiomyocytes and H9c2 cells are widely utilized as in vitro models in cardiovascular disease research. The H9c2 cell line, a subclone derived from embryonic BD1X rat cardiomyocytes, has been extensively employed globally in studies of various heart diseases. Hypertension‐induced ventricular remodeling is characterized by prominent cardiomyocyte hypertrophy. At the same time, decreased ventricular compliance leads to decreased diastolic function, which is the main pathophysiological mechanism of hypertension‐induced heart failure [20]. Over activation of the renin angiotensin system (RAS) is one of the important pathogenesis of hypertension. Related research reports [21] show that Ang II is one of the important effectors of the RAS system. Many studies [22, 23] have used Ang II to stimulate cardiomyocyte hypertrophy to establish a heart failure model in vitro. According to the relevant experimental studies, H9c2 cells and rat primary cardiomyocytes have no special significant difference in the hypertrophic effect of cardiomyocytes intervened by Ang II. Compared with rat primary cardiomyocytes, the H9c2 cell line is not only relatively simple in culture method but also has the relative advantages of easy access, passability, and high survival rate. Therefore, in the in vitro experiment, H9c2 cells were induced by Ang II with the objective to explore the molecular mechanism of RNA oxidation and its inhibitory enzymes involved in ventricular remodeling in vitro.

In this study, H9c2 cells were treated with step concentration Ang II (10^−8^, 10^−7^, 10^−6^ M) and step time Ang II (0, 12, 24, 36 h), respectively. The expression of 8‐oxoG in H9c2 cells was detected by cell immunofluorescence and ELISA. The change trend of 8‐oxoG expression in H9c2 cells of different groups was observed. To find out the optimal concentration and time of Ang II which can induce H9c2 cells to produce 8‐oxoG. First of all, when Ang II was fixed for 24 h, we found that the expression of 8‐oxoG was the highest when the concentration of Ang II was 10^−7^ M, so we took Ang II 10^−7^ M as the optimal concentration; then when the concentration of Ang II was fixed for 24 h, we found that the expression of 8‐oxoG was the highest, so we took Ang II 24 h as the optimal time. This experiment proved that Ang II could stimulate cardiomyocyte hypertrophy in vitro, which was the same as the research conclusion of Liu et al. [24]. In this experiment, the expression of 8‐oxoG in H9c2 cells was detected by immunofluorescence and ELISA, which can improve the accuracy of the experimental data. Therefore, in this study, a hypertensive heart failure cell model was established in vitro by stimulating H9c2 cells with 10^−7^ M Ang II for 24 h, which corresponds to the pathophysiological features observed in animal models and provides an experimental basis for further investigation into the underlying molecular mechanisms.

In this experiment, we compared the surface area of cardiomyocytes between the two groups under light microscope and found that the surface area of cardiomyocytes in Group S was larger than that in Group C. We also used PCR to detect ANP, BNP, and β‐MHC to evaluate the degree of cardiomyocyte hypertrophy and found that the values of the three in Group S were higher than those in Group C. The re‐expression of ANP [25], BNP [26], β‐MHC [27] genes in the process of cardiomyocyte hypertrophy has been recognized by the international community and is a marker gene of cardiomyocyte hypertrophy [28]. At the same time, detection of the expression of these three genes can not only determine whether cardiomyocyte hypertrophy occurs but also determine the degree of ventricular hypertrophy [28, 29]. Therefore, we conclude that the cardiomyocyte hypertrophy of the H9c2 cardiomyocyte model induced by Ang II is obvious, which verifies that the construction of the model is successful. This experimental result is consistent with the relevant international research [30, 31, 32]. Then, the content of 8‐oxoG in cardiomyocytes was determined by immunofluorescence assay and ELISA. We found that the C Group compared with the S Group: After 24 h stimulation with Ang II 10^−7^ M, the content of 8‐oxoG in H9c2 cells was higher than that in normal cells. Conclusion: in the model of H9c2 cardiomyocyte hypertrophy mediated by Ang II, the level of RNA oxidation increased significantly. Next, we detected the expression of MTH1 in H9c2 cardiomyocytes by WB and found that the expression level of MTH1 in Group S was significantly higher than that in Group C, which indicated that after Ang II stimulated cardiomyocytes, the level of RNA oxidation in cardiomyocytes was increased and the enzyme MTH1 that inhibited this effect was activated. Finally, we compared the activation levels of the ERK–MAPK pathway in the S Group and Group C and found that the activation level of the ERK–MAPK pathway was consistent with the animal experiment in vivo. After stimulation by Ang II, Raf1, MEK1/2, and ERK1/2 protein in Group S were activated step by step, which may be caused by the increase of RNA oxidation level and the increase of 8‐oxoG secretion. Many experimental studies have shown that [33, 34]. ERK signaling pathway is involved in the process of cardiomyocyte hypertrophy, which is consistent with the results of this experiment. The activation of the ERK signaling pathway protein in the cardiomyocyte hypertrophy group is also increased. In summary, the Ang II‐induced H9c2 cardiomyocyte model exhibited pronounced cellular hypertrophy, along with significantly elevated RNA oxidation levels, increased expression of the RNA oxidation inhibitor MTH1, and sequential activation of ERK–MAPK pathway proteins. These results demonstrate that alterations in RNA oxidation and its regulatory inhibitor are associated with the process of cardiomyocyte hypertrophy in vitro. Thus, our findings indicate that RNA oxidation and its inhibitor MTH1 contribute to the pathophysiological mechanisms underlying heart failure. Although the association between oxidative stress and MAPK signaling activation has been reported in cardiac pathologies [35, 36], prior studies have primarily focused on DNA oxidation or general ROS effects. Our study provides the first evidence that RNA‐specific oxidation (e.g., 8‐oxoG accumulation) serves as a distinct upstream trigger for ERK–MAPK activation in cardiomyocyte hypertrophy. Unlike previous works that attributed MAPK activation broadly to “oxidative stress,” we mechanistically demonstrate that:(1) 8‐oxoG directly activates Ras/Raf1/MEK/ERK cascades, independent of DNA damage; (2) RNA oxidation inhibitor MTH1 overexpression selectively suppresses this pathway; (3) The temporal sequence of 8‐oxoG accumulation preceding ERK phosphorylation suggests causality. This RNA‐centric perspective adds nuance to the oxidative stress theory of HF by highlighting transcriptome‐level damage as a specific actionable mechanism beyond genomic instability. In order to study how 8‐oxoG, a product of RNA oxidation, mediates cardiomyocyte hypertrophy, we constructed the over expression plasmid of RNA oxidation inhibitor MTH1 and transfected H9c2 cardiomyocytes with it, so as to establish a cell model with strong RNA oxidation defense ability. After overexpression of MTH1 in H9c2 cardiomyocytes, we found that the level of RNA oxidation in Group G was significantly lower than that in Group S, and the activation of the ERK–MAPK pathway was decreased, accompanied by the decrease of cardiomyocyte hypertrophy. This indicated that the ability of H9c2 cardiomyocytes to resist RNA oxidation was enhanced after increasing the expression of MTH1. After the stimulation of 8‐oxoG, the activation of the ERK–MAPK pathway proteins decreased correspondingly, which also verified that the molecular mechanism of RNA oxidation leading to cardiomyocyte hypertrophy may be the activation of the ERK–MAPK pathway mediated by 8‐oxoG. At the same time, we set up the empty plasmid transfection group of MTH1 and found that the plasmid had no significant effect on the experimental results, thus excluding the effect of the plasmid itself on RNA oxidation and cardiomyocyte hypertrophy. The results confirmed that RNA oxidation may lead to cardiomyocyte hypertrophy by activating the ERK–MAPK pathway in vitro.

Limitations of the experiments. In our experimental design, although ANP, BNP, and β‐MHC mRNA levels were quantified using real‐time PCR, the corresponding protein expression was not assessed. This omission was primarily due to the following considerations: (1) pathway focus: this study prioritized rapid transcriptional screening, where quantitative real‐time PCR efficiently captures mRNA‐level activation of hypertrophy‐related genes (ANP/BNP/β‐MHC), providing preliminary direction for subsequent mechanistic investigation; (2) practical constraints: protein extraction from cardiomyocytes requires synchronized cell lysis, concentration quantification, and electrophoresis. Combined with multiple intervention groups and time points (e.g., Ang II stimulation with MTH1 overexpression combinations), comprehensive Western blot analysis for all experimental groups was not feasible within the constrained experimental timeline; (3) methodological validation: previous studies [37] have demonstrated a strong positive correlation between mRNA and protein expression levels of ANP/BNP/β‐MHC in this H9c2 cell model (*r* > 0.850, *p* < 0.001). Therefore, we prioritized higher‐throughput qPCR for initial screening. Acknowledging the limitations of our in vitro model is crucial. The H9c2 cell line, while a valuable tool for mechanistic studies, is derived from rat embryonic myocardium and does not fully recapitulate the complex phenotype of adult primary cardiomyocytes, particularly in terms of contractile apparatus [38] and metabolic maturity [39]. Therefore, the extrapolation of these findings to human heart failure pathophysiology requires caution. However, the consistency between our cellular data and the results from our prior in vivo study significantly strengthens our conclusions. In that previous investigation using Dahl salt‐sensitive rats, we established that progressive RNA oxidation, quantified by elevated 8‐oxoG levels in urine and cardiac tissue, was intrinsically linked to the development of hypertension‐induced heart failure, correlating with functional deterioration and structural remodeling [19]. The concordance of the ERK–MAPK pathway activation and MTH1 upregulation across both the animal model and the H9c2 cell model suggests that the mechanism uncovered here is robust and operationally relevant in a more integrated physiological context. The animal data serve as a critical bridge, confirming that the phenomenon observed in cultured cells occurs in a living organism during the natural progression of the disease, thereby mitigating concerns solely reliant on the cell line model and enhancing the translational potential of our hypothesis.

In conclusion, we found that the expression of 8‐oxoG was significantly decreased and the activation of the Raf‐1–MEK1/2–ERK1/2 signaling pathway was decreased by the transfection of the MTH1 overexpression plasmid, which was accompanied by the decrease of cardiomyocyte hypertrophy. These findings establish a causal link between RNA oxidation and cardiomyocyte hypertrophy through the 8‐oxoG/ERK–MAPK axis. This mechanistic insight resonates with the broader research landscape. As highlighted by Kamihara et al. [40], while mitochondrial dysfunction, oxidative stress, and inflammation are established central themes in cardiac aging research—with diastolic dysfunction and fibrosis being key clinical manifestations—and while molecular mechanisms like telomere shortening and DNA damage are emerging foci, a significant gap remains in systematically understanding the role of RNA oxidation (e.g., 8‐oxoG accumulation) in pathological processes such as cardiomyocyte hypertrophy and heart failure. Our work provides a timely contribution to addressing this knowledge gap, suggesting that 8‐oxoG might induce cardiomyocyte hypertrophy by mediating the Raf‐1–MEK1/2–ERK1/2 pathway. Therefore, inhibition of RNA oxidation may interfere with the progress of ventricular remodeling, positioning RNA oxidation as a novel and specific mechanism within the established framework of oxidative stress in cardiac aging and HF.

## 5 Conclusions

In the Ang II mediated H9c2 cardiomyocyte hypertrophy model, the level of RNA oxidation and the expression of MTH1 were significantly increased, and the ERK–MAPK pathway was activated in sequence, which confirmed that the changes in RNA oxidation and its inhibitor were related to the process of cardiomyocyte hypertrophy in vitro. After overexpression of MTH1 in H9c2 cardiomyocytes, we found that the level of RNA oxidation was significantly decreased, and the activation of the ERK–MAPK pathway was decreased, accompanied by the decrease in cardiomyocyte hypertrophy, which further confirmed that RNA oxidation may lead to cardiomyocyte hypertrophy by activating the ERK–MAPK pathway.

## Author Contributions

Tong Liu: writing – original draft, data curation, validation, project administration, and analyzing the data. The remaining author participated in the discussion of cell experiments and data analysis. All the authors approved the submission.

## Funding

The authors have nothing to report.

## Conflicts of Interest

The authors declare no conflicts of interest.

## Data Availability

The data used to support the findings of this study are available from the corresponding author upon request.
